# Editorial Brevities

**Published:** 1887-08

**Authors:** 


### EDITORIALS AND MISCELLANEOUS.

### EDITORIAL BREVITIES.

SANDER & SONS' EUCALYPTI EXTRACT (EUCALYPTOL).—Apply to Dr. Sander, Dillon, Iowa, for gratis supplied reports on cures effected at the clinics of the Universities of Bonn and Griefswald.

MAGAZINE OF AMERICAN HISTORY.—We have before us the June number of the above magazine, which comes each month loaded with interesting historical matter and beautifully illustrated. This great historical monthly is without a rival in its special domain, and holds the highest rank in the current literature of the time. It overflows with varied and choice reading. Price, $5 00 a year in advance. Published at 743 Broadway, New York City.

AN IMPORTANT MEDICO-LEGAL DECISION.—We find the following in the *Sanitary News:*

In the Superior Civil Court at Boston, recently, a mother and her four children individually sued the landlord to recover damages for sickness contracted because of the poor sanitary condition of the house and in care of the family during their sickness from diphtheria. Damages in each case were awarded, the mother receiving $1,600, and the children $700, $300, $250 and $200, respectively. The case is valuable as a precedent.

DR. ODIORNE DEAD.—We regret to hear of the death of Dr Jas. Odiorne, a highly esteemed and worthy member of the profession in Johnson City, Texas. His death was occasioned by the accidental explosion of a can of alcohol in a drug store, from which the doctor was so seriously burned as to cause his death. And so our brethren pass away.

Dr. J. R. Reeves, who kept office in the same building, sustained a heavy loss in the destruction of his medical books and surgical instruments.

